# New Insights into Diabetic Kidney Disease: The Potential Pathogenesis and Therapeutic Targets

**DOI:** 10.1155/2017/3945469

**Published:** 2017-11-08

**Authors:** Wei Jing Liu, Jochen Reiser, Tae Sun Park, Zhangsuo Liu, Shuta Ishibe

**Affiliations:** ^1^Key Laboratory of Chinese Internal Medicine of Ministry of Education and Beijing, Dongzhimen Hospital Affiliated to Beijing University of Chinese Medicine, Beijing, China; ^2^Rush University, Chicago, IL, USA; ^3^Chonbuk National University Medical School, Jeonju, Republic of Korea; ^4^The First Affiliated Hospital of Zhengzhou University, Zhengzhou, China; ^5^Yale University School of Medicine, New Haven, CT, USA

Diabetic kidney disease (DKD) is an established complication of long-term inadequate glycemic control with significant medical and social burden and has become the single most frequent cause of end-stage renal disease (ESRD). In developed countries, DKD is the leading cause of ESRD, accounting for 44% of cases in the United States [1]. In Asia, the corresponding DKD is about 40% of total in Japan [2] and 48% in Korea [3] and the incidence of DKD in hospitalized diabetic patients has reached 34.7% in China [4]. Many patients with DKD unfortunately progress to ESRD as therapeutic regimens are still lacking, although tight glycemic and blood pressure control may slow its progression.

It is well known that the pathophysiology of DKD remains complex and multifactorial [5]. However, progress has been made in understanding the molecular mechanisms in DKD. For example, while DKD is considered a microvascular complication of diabetes, growing evidence indicates that podocyte loss and epithelial dysfunction link many initial events of DKD. Recent evidence suggests the autophagic inactivation and accelerated senescence in renal tubular cells play a fundamental role in the progression of DKD [6]. In view of these findings, understanding the underlying pathophysiology of DKD is critical to unearth additional treatment modalities and strategies. This special issue contains several papers including original research articles and reviews on this topic to help in unraveling the potential pathogenesis and identifying novel therapeutic targets of DKD.

W. Cui et al. demonstrated the therapeutic effects of Nrf2 activation on DKD and concluded that modulating Nrf2/ARE pathway through different mechanisms provides new approaches for future clinical research and the treatment of DKD. But in order to avoid serious adverse events, the potential side effects of Nrf2 activation will need to be monitored during the trial. J. Sun et al. concisely summarized the evidence that epigenetic histone modifications have a significant effect in modulating renal fibrotic and ECM gene expression induced by TGF-*β*1, as well as its downstream profibrotic genes in the state of diabetes and hyperglycemia. Histone modifications are also implicated in renal fibrosis through its ability to regulate the EMT process triggered by TGF-*β* signaling. R. R. Dande et al. demonstrated the link between uPAR and suPAR in the clinical manifestations of DKD, which may enlighten us our understanding of the pathogenesis of DKD and provide avenues for the treatment of the disease. H. Dai et al. provided a review highlighting the signaling pathways in podocytes that underlie the development of albuminuria in DKD.

H. Zhang et al. reported that top 10 hub genes were selected from the constructed PPI network of total 355 differentially expressed genes (DEGs) identified by microarray analysis of db/db mice, including Ccnb2 and Nr1i2, which remained largely unclear in DKD. The pathway enrichment analysis suggested that biological oxidation, bile acid metabolism, and steroid hormone synthesis were the 3 major significant pathways that may contribute to DKD pathogenesis.

Using HKC8 cells and STZ-induced mice, S.-Y. Lee et al. demonstrated that increases in PGC1*α* activity could improve diabetic tubulopathy by modulating mitochondrial dynamics and autophagy in vivo and in vitro. They reported that increasing PGC1*α* activity might improve functional mitochondrial mass in HKC8 cells in high-glucose condition and in renal proximal tubular cells and reverse the changes in Drp1, Mfn1, and LC3-II protein expression in a high-glucose environment. In vivo, increased PGC1*α* activity resulted in low ROS production and reduced apoptosis by normalizing mitochondrial life cycles, mitigated albuminuria and renal histopathology, and decreased expression of TGF*β*1 and *α*-SMA in the kidneys of diabetic mice.

H. Zhu et al. reported a novel pattern of glycosylation of urinary protein as a new potential diagnosis biomarker for DKD. By using urinary protein microarray, they found that along with the development of DKD, the levels of Sia*α*2-6Gal/GalNAc recognized by SNA exhibited significantly increased tendency and (GlcNAC)_2-4_ may be a potential biomarker to differentiate patients with DKD from non-DKD.

Shen-Yan-Fang-Shuai Formula (SYFSF) is an effective traditional Chinese formula to treat DKD, yet the molecular mechanisms on renoprotection are largely unknown. J. Lv et al. interrogated the renoprotective effects in a diabetic rat model and in high-glucose cultured mesangial cells. They reported that SYFSF downregulated the expression of MCP-1, TGF-*β*1, collagen IV, and fibronectin. The renoprotective effects are potentially attributable to an inhibition of inflammatory responses and extracellular matrix (ECM) accumulation mediated by TNF-*α*/NF-*κ*Bp65 signaling pathway.

The renoprotection of sulodexide (SDX) remains controversial. Y. N. Liu et al. determined whether SDX has renoprotection at an early or late stage of DKD. They found that in STZ-induced diabetic rats, SDX may prevent the progression of DKD if initiated early, attributing the mechanism to the upregulation of Klotho expression, which inhibited the tubulointerstitial injury induced by oxidative stress in diabetes.

In conclusion, the reviews and original basic science manuscripts in this special issue provide a thought-provoking avenue to understand the pathogenesis and therapeutic targets in DKD.
